# Molecular Integration in Adenosine Heteroreceptor Complexes Through Allosteric and De-Phosphorylation (STEP) Mechanisms and its Role in Brain Disease

**DOI:** 10.3389/fphar.2021.781381

**Published:** 2022-01-05

**Authors:** Dasiel O. Borroto-Escuela, Luca Ferraro, Kjell Fuxe

**Affiliations:** ^1^ Department of Neuroscience, Karolinska Institutet, Stockholm, Sweden; ^2^ Department of Life Sciences and Biotechnology, University of Ferrara, Ferrara, Italy

**Keywords:** adenosine receptors, adenosine heteroreceptor complexes, dopmaine heteroreceptor complexes, mu-opioid heteroreceptor complexes, G protein coupled receptors (GPCR), receptor-receptor interactions, striatal-enriched protein tyrosine phosphatase, allosteric modulation

## Introduction

Dysfunction of adenosine heteroreceptor complexes can contribute to mental disorder development (Borroto-Escuela et al., 2018a; Beggiato et al., 2021; Merighi et al., 2021). Furthermore, a molecular basis for learning and memory was proposed to be formed through reorganization of available adenosine homo- and heteroreceptor complexes as to structural functions and/or by resetting the multiple allosteric receptor-receptor interactions in these complexes. Based on this evidence, this Opinion article is focused on the underlying relevance of adenosine heteroreceptor complexes in the brain and their integrative mechanisms at the molecular level, involving allosteric receptor-receptor interactions and dephosphorylation mechanisms through Striatal-Enriched Protein Tyrosine Phosphatase (STEP) (Franco et al., 2020b; Yasuda, 2020; Borroto-Escuela et al., 2021a; Barresi et al., 2021; Borroto-Escuela et al., 2021b; Beggiato et al., 2021; Domenici et al., 2021).

This enzyme is a tyrosine phosphatase specific to the brain and its substrate is represented by a vast network of synaptic and extra synaptic proteins (Lombroso et al., 1993; Won et al., 2019). Several splice variants exist, namely STEP_61,_ STEP_46,_ STEP_38_, and STEP_20_. However, only STEP_46_ is enriched in the striatum and STEP_61_ shows a widespread distribution with high densities e.g., in cerebral cortex and hippocampus (Won and Roche, 2021). STEP has several functions through it dephosphorylation, via its enzymatic activity, of synaptic and extra synaptic proteins including kinases and glutamate receptors like NMDARs and AMPARs producing synaptic downregulation (Zhang et al., 2008; Won et al., 2019; Won and Roche, 2021). STEP dysfunction may therefore lead to disturbances in synaptic plasticity necessary for cognition. How STEP and adenosine A2A heteroreceptor complexes can modulate between each other and have a role in molecular integration of adenosine signal in the brain, is the focus of this opinion article.

## Understanding the Mechanisms for A2AR Mediated Activation of STEP: Involvement of A2AR Heterocomplexes

Robert Yasuda (Yasuda, 2020) wrote a highly interesting Editorial on STEP (Striatal -enriched protein tyrosine phosphatase in neuronal cells and its modulation by adenosine A2A receptors (Mallozzi et al., 2020), published in Journal of Neurochemistry. As discussed by Yasuda (2020), cocaine induced increases in brain adenosine levels (Kubrusly and Bhide, 2010) can help explain how cocaine can activate the A2AR which leads to increases in the dephosphorylation activity of STEP on synaptic glutamate receptors with associated depression of synaptic activity (Mallozzi et al., 2020). These results are supported by the early work on cocaine by Chiodi et al. (2014). Also by the observation that A2AR mediated synaptic depression was counteracted not only by an A2AR antagonist but also by a dopamine D2R antagonist. This can be explained by the fact these two receptors are primarily located in the dorsal and ventral striatal pallidal GABA neurons mediating motor inhibition and antireward, respectively (Borroto-Escuela et al., 2021a; Borroto-Escuela et al., 2021b).

**FIGURE 1:**
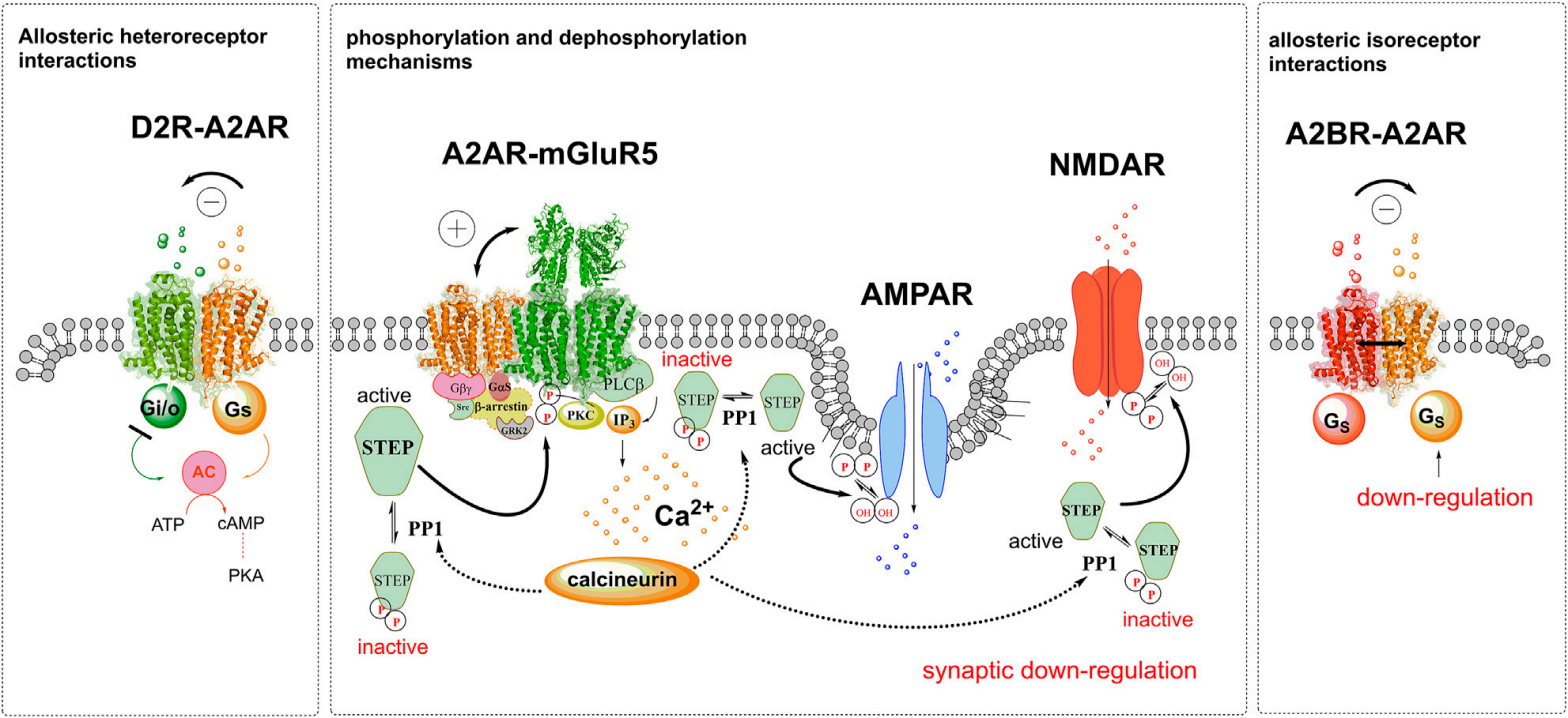
Illustration of allosteric receptor-receptor interactions is shown in D2R-A2AR, A2AR-mGluR5 and A2BR-A2AR heterocomplexes and of activation of STEP through enhanced mGluR5 protomer signaling. To the left side, the A2AR agonist activation of the A2AR protomer leads to allosteric inhibition of the inhibitory Gi/o mediated signaling of the D2R, reducing the inhibition of the adenylate cyclase (AC). To the right side the constitutive activity (black line) of the A2BR leads to an allosteric downregulation of the Gs mediated activation of the A2AR. In the center the facilitatory allosteric interactions in the A2AR-mGluR5 heterocomplex is illustrated. It involves enhanced activity of the Gq mediated signaling of the mGluR5 with enhanced PLC-beta signaling leading to increased PKC activity with the enhanced formation IP3 increasing intracellular calcium levels. As a result, the calcineurin-PP1 pathway becomes activated and dephosphorylates STEP which causes its activation. The activated STEP can then dephosphorylate NMDAR and AMPAR with a return of the hydroxyl (OH) groups and a synaptic down regulation takes place. Also, mGluR5 binds STEP and becomes inactivated through its de-phosphorylation. In this way the mGluR5 may no longer be activated which becomes true also for Calcineurin and STEP activity becomes reduced with the return of synaptic activity.

The D2R activation markedly inhibits the firing of these two types of neurons via its inhibitory Gi/o coupling of the D2R, causing increases in motor activity and reductions of anti-reward. The striatal-pallidal GABA neurons are enriched in A2AR-D2R heterocomplexes in which the activated A2AR protomer strongly blocks the D2R protomer mediated signaling through allosteric inhibition of the D2R protomer (Borroto-Escuela et al., 2021b) (Figure 1). It should be noted that the work of the Italian group in 2020 takes place in striatum, which may be further defined as to the possible inclusion also of the ventral striatum or if it only involved the dorsal striatum. Robert Yasuda indicates the relevance of these antagonistic A2AR-D2R interactions in the A2AR-D2R heterocomplexes of the ventral striatal-pallidal GABA neurons for the A2AR mediated inhibition of cocaine self-administration (Borroto-Escuela et al., 2021a; Borroto-Escuela et al., 2021b). Disruption of this receptor complex with an interface interfering peptide in fact blocks the A2AR mediated inhibition of cocaine self-administration (Borroto-Escuela et al., 2018b). This A2AR-D2R mechanism lacks the pathway to induce STEP activation.

The first indication that cocaine self-administration increases the enzymatic activity of STEP was obtained in the prefrontal cortex in 2013 (Sun et al., 2013), leading to diminished expression of GluN. The following year it was found that cocaine in micromolar concentrations diminished excitatory post-synaptic transmission in the dorsal striatum that involved STEP activation (Chiodi et al., 2014). It was pointed out that the blockade of the synaptic depression by an A2AR antagonist, indicated the participation of A2ARs (Yasuda, 2020). These results were additionally supported by the observations that A2AR overexpression in the brain, especially in striatum, could produce increased STEP activity (Mallozzi et al., 2020). Yasuda in his editorial (Yasuda, 2020) searched for a possible mechanism by which A2AR activation could increase STEP activity. He discussed in a clear way how STEP could be activated in the striatal-pallidal GABA pathway by increased A2AR functionality, capable of producing increases in intracellular calcium levels. It involved activation of phospholipase C (PLC) followed by IP3 induced increases in intracellular calcium levels leading to calcineurin/PP1 activation causing dephosphorylation of STEP which induces its activation (Yasuda, 2020; Domenici et al., 2021).

## Understanding the Role of the A2AR-mGluR5 Heterocomplexes in STEP Activation

In the striatal-pallidal GABA neurons, in which A2AR-D2R heterocomplexes are enriched (Borroto-Escuela et al., 2021a) there also exist A2AR-mGluR5 and A2AR-mGluR5-D2R heterocomplexes (Ferre et al., 2002; Ciruela et al., 2005; Fuxe et al., 2008; Cabello et al., 2009; Borroto-Escuela et al., 2017). It is known that A2AR may allosterically enhance the mGluR5 signaling and its Gq/11 mediated signaling coupled to activation of PLC leading to increases in calcium levels and activation of calcineurin and activation of STEP as discussed (Yasuda, 2020; Borroto-Escuela et al., 2021a; Domenici et al., 2021).

It is also of high interest that STEP can bind to mGluR5, showing a special role of mGluR5 capable not only to activate STEP but also by its own ability to bind and be downregulated by this phosphatase (Won et al., 2019) (Figure 1). Furthermore, STEP can produce downregulation of AMPA receptors upon mGluR1/5 receptor activation (Zhang et al., 2008).

Yasuda (Yasuda, 2020) also discussed theA2AR overexpression model used in the Mallozzi et al. (Mallozzi et al., 2020) paper to establish the strong activation of A2AR leading to increased STEP activity in striatum and hippocampus counteracted by an A2AR antagonist which failed to have effects in wild-type animals. It seems possible that upon A2AR overexpression a marked increase in the density of the A2AR-mGluR5 complexes can develop. These events can contribute to the strong activation of STEP leading to the downregulation of excitatory synaptic signaling with substantial negative consequences for learning and memory (Gimenez-Llort et al., 2007; Borroto-Escuela et al., 2015; Hamor et al., 2020; Yasuda, 2020).

## The Balance of A2AR-mGluR5 and A2AR-D2R Heterocomplexes in Cocaine Self-Administration

Substantial differences were observed in ventral compared to dorsal striatum regarding the results obtained in effects of cocaine self-administration on A2AR-mGluR5 and A2AR-D2R heterocomplexes (Borroto-Escuela et al., 2017). In contrast to the dorsal striatum, there was a clear-cut and significant increase of the A2AR-D2R heterocomplexes and a trend for an increase for the A2AR-mGluR5 heterocomplexes in nucleus accumbens shell (Borroto-Escuela et al., 2017). These results are compatible with a preferential allosteric enhancement by the A2AR agonist of the mGluR5 protomer signaling in this complex located in the nucleus accumbens shell versus the dorsal striatum. It should lead to increased activation of STEP as discussed, associated with an excitatory synaptic downregulation. In parallel, the A2AR agonist will strongly inhibit the D2R protomer signaling in this region of the nucleus accumbens. The significance of these two allosteric events induced by the A2AR agonist leading to inhibition of the D2R protomer and to synaptic downregulation of parts of the ventral striatal-pallidal GABA antireward neurons remains to be determined. However, differences in their time dynamics can play a role with the A2AR mediated inhibition of the D2R signaling being more long-lasting versus more dynamic changes in the excitatory synaptic transmission.

The absence of increases in A2AR-mGluR5 heterocomplexes in the caudate putamen in cocaine self-administration opened the existence for another A2AR mechanism mediating the activation of STEP. It was proposed that the A2AR-FGFR1 heterocomplex discovered in the dorsal striatum (Flajolet et al., 2008) could be involved in mediating the STEP activation (Borroto-Escuela et al., 2021a). It was suggested that a new panorama of transcriptional proteins could be formed upon co-activation of this receptor complex leading to formation of proteins with phosphatase activity taking over the function of e.g., protein-tyrosine-phosphatase 1 (PPT1) (Borroto-Escuela et al., 2021a).

## Understanding the Role of A2AR Heterocomplexes in STEP Actions in Alzheimer’s Disease and Parkinson’s Disease

It is known that STEP is elevated in Alzheimer’s disease and produces significant increases in synaptic down-regulation through dephosphorylation and internalization of e.g., N-methyl-d-aspartate receptor (NMDAR) and α-amino-3-hydroxy-5-methyl-4-isoxazolepropionic acid receptor (AMPAR) (Snyder et al., 2005; Zhang et al., 2008; Zhang et al., 2010). It is of special interest that long-term activation of the NMDAR through stimulation of the calcineurin/PP1 intracellular pathway can dephosphorylate STEP leading to its activation in the striatum (Valjent et al., 2005). It can represent a significant feed-back to counteract an overactivation of the NMDAR which can lead to excitotoxicity (Mahaman et al., 2021).

Recently it was proposed the existence of putative A2AR-D2R-NMDAR heterocomplexes (Borroto-Escuela and Fuxe, 2019). It should be underlined that D2R can also form complexes with the NMDAR in which allosteric inhibitory D2R-NMDAR receptor subtype 2B (NR2B) interactions exist (Liu et al., 2006). It was also possible to demonstrate with BRET and *in situ* PLA the existence of NMDAR-A2AR heteroreceptor complex in HEK and microglia cells (Franco et al., 2020a). The results from Franco et al. (2020a) may indicate that the A2AR expression can increase the function of the NMDAR signaling in microglial cells, while the activation of the NMDAR can block the A2AR function. Furthermore, antagonistic allosteric receptor-receptor interactions may exist within the A2AR-NMDAR heteromer. Based on these findings it was proposed that A2AR antagonists have a potential to counteract neurodegeneration in Alzheimer’s disease.

In the paper of Borroto-Escuela and Fuxe (Borroto-Escuela and Fuxe, 2019), it was suggested that the alpha-synuclein monomer (alpha conformation) may bind to the A2AR protomer of the A2AR-D2R-NMDAR heterocomplex. The alpha-synuclein monomer may enhance the A2AR protomer activation and thus its allosteric inhibition of the D2R protomer signaling. As a consequence, the allosteric inhibition by the D2R protomer of the NMDAR protomer is reduced (Liu et al., 2006) and NMDAR function can become strongly enhanced with increased calcium influx over its ion channels.

This leads to coupling to nitric oxide production and toxicity. The calcium influx can then via the calcineurine/PP1 pathway de-phosphorylate STEP leading to its activation. In this way the STEP can bring down NMDAR signaling and induce its internalization and block the excitotoxicity caused by excessive activation of the NMDAR. These results give increased implications for using A2AR antagonists in the treatment of Parkinson’s disease (Borroto-Escuela and Fuxe, 2019; Perez de la Mora et al., 2020). Finally, it should be considered that beta sheet intermediates of alpha-synuclein peptides may bind to the intracellular loops and C terminals of the GPCR protomers of the heteroreceptor trimeric complex and modulate its signaling and its response to activated STEP.
